# The application of magnetic susceptibility separation for measuring cerebral oxygenation in preterm neonates

**DOI:** 10.1038/s41390-025-03966-6

**Published:** 2025-03-19

**Authors:** Thomas Gavin Carmichael, Alexander Rauscher, Ruth E. Grunau, Alexander Mark Weber

**Affiliations:** 1 Integrated Sciences, The University of British Columbia, Vancouver, BC Canada; 2 BC Children’s Hospital Research Institute, The University of British Columbia, Vancouver, BC Canada; 3 Physics and Astronomy, The University of British Columbia, Vancouver, BC Canada; 4 Pediatrics, The University of British Columbia, Vancouver, BC Canada

## Abstract

**Background:**

Quantitative susceptibility mapping (QSM), a magnetic resonance imaging (MRI) modality sensitive to deoxyhemoglobin, is a promising method for measuring cerebral oxygenation in human neonates. Paramagnetic sources, like deoxyhemoglobin, however, can be obscured by diamagnetic sources such as water and myelin. This study evaluated whether QSM images, or isolated paramagnetic components, are more accurate for measuring oxygenation of cerebral veins of preterm neonates, and explored oxygenation differences between the major cerebral veins.

**Methods:**

19 preterm neonates were scanned on at term equivalent age on a 3T MRI using a multi-echo susceptibility-weighted imaging sequence. Susceptibility values were calculated from QSM images to determine oxygen saturation (SvO_2_) in the superior sagittal sinus (SSS) and central cerebral veins (CCV). The paramagnetic components of QSM images were isolated, and SvO_2_ values were recalculated.

**Results:**

The mean SvO_2_ values from QSM were 72.4% (SD, 3.4%) for the SSS and 68.7% (SD, 3.5%) for the CCV. SvO_2_ values for paramagnetic components were 58.1% (SD, 7.3%) for the SSS and 57.7% (SD, 7.0%) for the CCV.

**Conclusion:**

While paramagnetic component decomposition yielded SSS values closer to those found in the literature, it increased variability. No significant oxygenation differences were found between the SSS and CCV, contrasting with prior studies.

**Impact:**

- This study evaluated the use of QSM and its paramagnetic components to measure cerebral oxygenation in neonates.
- By comparing susceptibility-derived oxygen saturation (SvO_2_) in the superior sagittal sinus (SSS) and central cerebral veins (CCV), it adds to the field of neonatal cerebral oxygenation measurement.
- Decomposing QSM into paramagnetic components shows potential for improving SvO_2_ accuracy, particularly in the SSS, though variability remains a challenge.
- The results suggest no significant oxygenation difference between the SSS and CCV, contrasting with previous findings, indicating a need for further research on neonatal venous oxygenation.

## Introduction

With advances in neonatal medical care, more infants born preterm are surviving into childhood.^1^ These children are at high risk of acquiring adverse neurodevelopmental outcomes when compared to their term-born peers.^2^ Irregularities in early cerebral oxygen levels have been identified as a potential source of such delays, where too little oxygen provided during NICU care can result in white matter injury, while too much oxygen can result in reduced cortical connectivity.^3^ As such, being able to precisely, accurately, and non-invasively measure cerebral oxygenation is necessary for understanding and improving neurodevelopmental outcomes in preterm neonates.

Unfortunately, there exist many challenges in measuring cerebral oxygen metabolism in neonates. Cerebral metabolic rate of oxygen (CMRO_2_) using oxygen-15 positron emission tomography (PET),^4^ has been measured in infants,^5^ and is considered the gold standard. However, this method is invasive, requiring ionizing radiation, which limits its suitability for neonates. A less invasive option for evaluating brain hemodynamics is near-infrared spectroscopy (NIRS), which uses the attenuation of near-infrared light (~650–950 nm) as it passes through biological tissue.^6^ Deoxygenated and oxygenated hemoglobin absorb this light differently, allowing NIRS to estimate changes in deoxyhemoglobin and oxyhemoglobin^7^ and thus provide an estimate of cerebral venous oxygen saturation (SvO_2_). While NIRS offers the advantage of being non-invasive and continuous bedside monitoring, it is limited to regional assessments where the probe is placed and is sensitive only to superficial brain tissue due to the shallow penetration depth of near-infrared light.^8^

For the preceding reasons, non-invasive MRI-based techniques are actively being explored to assess regional and whole-brain blood oxygenation. While MRI-based methods have been developed for adults,^9–11^ their application in neonates is only beginning to be explored.^12–16^ This delay is likely due to the unique challenges posed by neonates, including their smaller anatomies, distinct hemodynamic profiles, susceptibility to motion artifacts, and the difficulties associated with recruiting this population for research. These methods have almost all relied on T2 relaxation to estimate CSvO_2_^12–14,16^ with the exception of^15^, which used susceptometry.^17^ One limitation of these T2 relaxation methods, however, is the fact that SvO_2_ is often measured using a single imaging slice, averaging values across several voxels, and only in the superior sagittal sinus (SSS). In the case of ref.^15^ they obtained regional and whole-brain data, but with thick slices (5 mm), and still only estimated CSvO_2_ in the SSS. An alternative MRI method using quantitative susceptibility mapping (QSM) has been proposed, which can measure SvO_2_ regionally and across the whole-brain at high resolution (<1mm^3^ per voxel).^18^ However, this method left room for improvement, as it removed the SSS (averaging CSvO_2_ across the internal veins), and required an arbitrary threshold value of 0.15 ppm in order to acquire realistic results.^18^ Furthermore, QSM tends to underestimates parametric components due to the inclusion of diamagnetic tissue, and vice versa, as the opposing magnetic susceptibilities effectively subtract from one another.^19^

In the present study, we set out to determine whether decomposing the QSM image into its paramagnetic and diamagnetic components would allow for a more accurate assessment of SvO_2_ in the central cerebral veins (CCV) of a cohort of preterm neonates. We also had a secondary aim of preserving the SSS vessel in our QSM images and using this data to determine whether a difference in oxygenation existed between the SSS and the CCV.

## Methods

The study was approved by the Clinical Research Ethics Board at the University of British Columbia and Children’s & Women’s Hospital (H21-00655), and written informed consent was obtained from the parent/guardian for each infant.

### Study population

Participant data comes from a previous study^20^. Participants consisted of preterm neonates born between 25- and 31-weeks gestational age (GA) who were admitted to the level III NICU at BC Women's Hospital. Recruitment took place over a span of one year, from February 2021 to January 2022, facilitated by a dedicated research nurse. Parents of eligible infants were approached by the research nurse prior to discharge from the NICU to explain the study objectives and seek their consent for participation. Infants meeting the criteria for inclusion were scanned for the study if they had already been discharged from the NICU, were in stable condition, and had reached a term equivalent age of 37 to 44 weeks GA. However, certain exclusion criteria were applied to ensure the homogeneity and integrity of the study sample: infants were excluded if there was clinical evidence of a congenital malformation or syndrome, a TORCH infection, or ultrasound evidence of large parenchymal hemorrhagic infarction (>2 cm, Grade 4 intraventricular hemorrhage).

### Image acquisition

MR imaging was performed on a 3.0 Tesla General Electric Discovery MR750 scanner (scanner software version DV26.0_R03) equipped with a SREE Medical Systems (Cleveland, OH) single-channel neonatal head coil (Table 1). The scans were conducted at the BC Children’s MRI Research Facility. Prior to the scanning procedure, subjects were carefully prepared by a research nurse. Swaddling and feeding were used to ensure the comfort and cooperation of the subjects during the scan. Importantly, no sedatives or invasive markers were utilized throughout the procedure. Subjects were placed within a specially designed SREE Medical Systems MRI compatible incubator, which facilitated both safety and motion minimization. Molded foam was strategically positioned around the head and body within the incubator to further restrict subject movement. To protect against potential hearing damage, earplugs were employed during the scanning process. Additionally, a pulse oximeter was affixed to the subject’s foot to monitor arterial oxygen saturation and heart rate throughout the scan.

**Table 1:** Technical parameters for MR imaging pulse sequences.

	T1w	T2w	pcASL	SWI
Sequence	3D FSPGR	3D CUBE	Multi-shot 3D fast spin-echo	3D spoiled GRE flow-compensated
Acquisition plane	Coronal	Sagittal	Axial	Axial
Phase-encoding direction	Left-Right	Posterior-Anterior	Posterior-Anterior	Left-Right
TR (ms)	7.74	2300	4680	30.9
TE (ms)	2.97	66.29	10.55	5 echoes; first echo: 5; echo spacing: 5.24
Flip angle	12°	90°	111°	20°
FOV (cm)	20	20	24	25
Acquisition matrix	512 × 512	256 × 256	128 × 1 28	256 × 256
In-plane resolution (mm)	0.39 × 0.39	0.78 ×0.78	1.875 × 1.875	0.977 × 0.977
Slice thickness (mm)	1	1	4	2, reconstructed to 1 with zero filling (ZIP2)
Number of slices	126	106	50	92
Additional parameters	n/a	n/a	1450 ms label period; 2025 ms pulse label; 24 control-label pairs	n/a
Scan duration	4 min 39 s	5 min 1 s	5 min 26 s	5 min 29 s

*T1w* = T1-weighted, *T2w* = T2-weighted, *pcASL* pseudo-continuous arterial spin labeling, *SWI* susceptibility weighted imaging, *FSPG* fast spoiled gradient echo, *CUBE* General Electric name of sequence, not an acronym, *GRE* gradient echo, *ZIP2* through-plane zero filling interpolation.

The MRI scan protocol comprised of the following sequences (plane of acquisition in parentheses): a T1-weighted scan (coronal), a T2-weighted scan (sagittal), a pseudo-continuous arterial spin labeling (ASL) scan^21^ (axial), a multi-echo susceptibility-weighted imaging scan^22^ (axial), and a diffusion-weighted imaging (DWI) spin-echo echo planar imaging (EPI) sequence (axial). The DWI sequence was not used for the present study.

### Image analysis

**Fig. 1:**
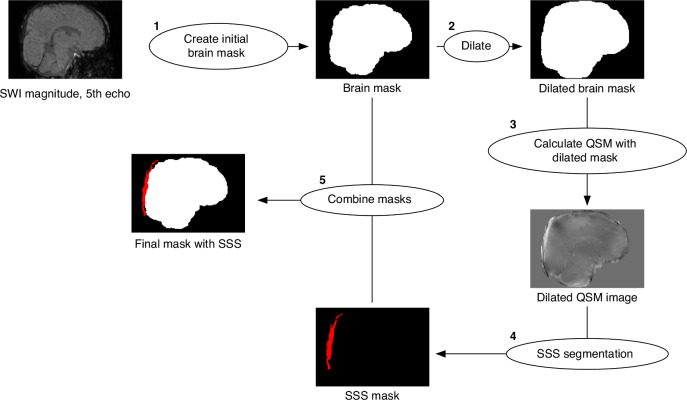
Pipeline for generating subject-specific brain masks that include the superior sagittal sinus (SSS). Initial steps involved (1) creating a brain mask from the magnitude of the fifth echo of the susceptibility weighted scan. Subsequently, the brain mask is dilated and then (2) utilized in conjunction with a quantitative susceptibility mapping (QSM) script to generate a preliminary QSM image. Further refinement involved (3) segmenting the SSS from the QSM image manually to create a tissue mask of the SSS region. Finally, (4) the vascular mask of the SSS is integrated with the initial brain mask, forming the comprehensive brain mask essential for obtaining susceptibility data that includes the SSS.

The raw DICOM files acquired from the scanning procedure were converted to NIfTI (Neuroimaging Informatics Technology Initiative) format using Chris Rorden’s dcmniix tool.^23^ SWI magnitude data files were then used to create subject-specific brain masks that would not erode the SSS during QSM processing, an issue faced by our group in the past^18^. A step-by-step summary of the pipeline used is shown in Fig. 1.

First, the fifth echo (TE = 25.96 ms) SWI magnitude file was processed using FSL’s (v. 6.0.7.3)^24^ fslroi, fslmaths, and bet^25^ to create a preliminary brain mask, similar to our previous efforts, which does not contain the SSS. The last echo is used to generate the brain mask as it reliably removes artifacts from air-tissue and bone-tissue interfaces, e.g., sinuses, without the need for manual erosion. The reason for this is at longer echo times, tissues with rapid signal decay (such as bone, air, and sinuses) lose their MRI signal due to dephasing caused by greater magnetic field inhomogeneities. Fslroi was used to isolate the fifth echo of the magnitude data, which was then squared using fslmaths and the option -sqr. Squaring the magnitude image was found to dramatically improve subsequent brain extraction. The resulting image was then used to create the preliminary brain mask using bet with the options -m and -R. The former flag generated a binary brain mask, while the latter performed a more robust brain center estimation. The brain mask was then dilated by 7 voxels using fslmaths and the options -kernel boxv and -dilM in order for the dilated mask to contain the SSS (along with unwanted tissue as well). This mask was then used, along with the phase images, in a MATLAB script for QSM calculation from Christian Kames^26^ to produce a preliminary QSM image that contained the SSS, albeit with fairly low signal-to-noise ratio and other unwanted tissue. Given the high contrast in voxel intensity between the SSS and surrounding tissue, the select by intensity tool in FSLeyes^27^ was then used to segment the SSS from the QSM image and create a 3D mask of the selected region. Using fslmaths and the options -add and -bin, the SSS mask was then combined with the original brain mask of the fifth echo. This resulted in a brain mask that contained only brain and SSS signal. Finally, this mask was used in a final QSM post-processing step to create a QSM image that includes the SSS while maintaining a high signal-to-noise ratio, making it suitable to obtain accurate susceptibility values.

**Fig. 2:**
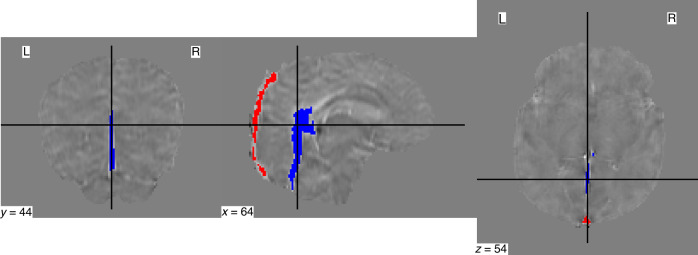
Sample venous masks. A sample superior sagittal sinus (red) and central cerebral vein mask (blue) displayed in coronal, sagittal, and axial view. The QSM image is used as the underlay. y, x and z values represent the slice number in each plane (coronal, sagittal, and axial, respectively).

STI Suite (v. 3.0),^28^ was used to process the final QSM images as it produced the images with the least amount of artifacts (based on a visual assessment by the authors) without eroding the SSS. The finalized brain mask along with all five echoes of the magnitude and phase images were used in STI Suite along with the following parameters: 0.9766 ×0.9766 ×1 mm^3^ voxel size, 5 ms TE1, 5.24 ms $\varDelta$TE, and 77.4 ms sum TE, B0 strength = 3, and B0 direction = [0, 0, 1]. The 3D GRE data option was selected for the phase processing stage, and STAR-QSM was selected for the QSM stage. STAR-QSM outputs a single QSM map for each echo (i.e. five total). The last three echoes of the QSM maps were then averaged to create the final QSM image using fslmaths, as the accumulation of phase due to susceptibility is small in early echoes and artifacts dominate the phase.^29^ Finally, the ‘select by intensity’ tool in FSLeyes was then used to semi-automatically make vascular masks of the SSS and CCV from each subject’s QSM image (Fig. 2). The vascular masks were used to calculate the mean susceptibility of each subject’s SSS and CCV from their QSM image with fslstats.

**Fig. 3:**
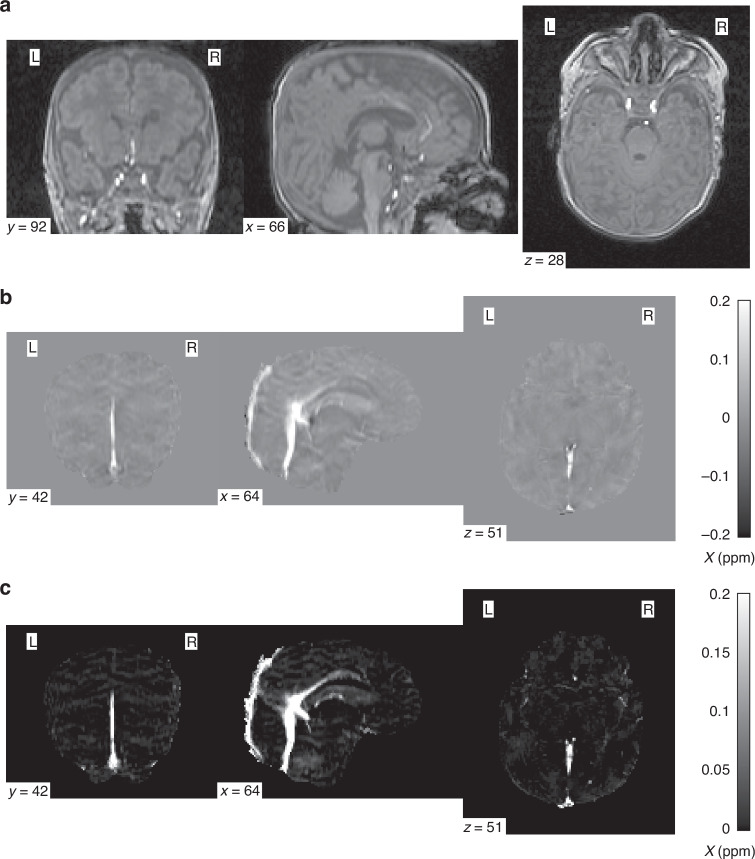
An example of subject imaging data. A sample coronal, sagittal, and axial slice is displayed for each image. **a** The 1st echo of the magnitude susceptibility weighted imaging sequence; **b** the final quantitative susceptibility mapping image; and (**c**) the paramagnetic component isolated from the quantitative susceptibility map. The color bars in (**b**) and (**c**) indicates the range of susceptibility $\chi$ values in parts per million. y, x, and z values represent the slice number in each plane (coronal, sagittal, and axial, respectively).

To isolate the paramagnetic component of subjects’ QSM data, the $\chi$-separation toolbox^30^ from the Laboratory for Imaging Science and Technology was used. All five echoes of each subject’s magnitude and phase SWI data were used along with the following parameters: 0.9766 × 0.9766 ×1 mm^3^ voxel size; TE (ms) = [5, 10.24, 15.48, 20.72, 26.96]; $\varDelta$TE (ms) = 5.24; B0 strength = 3; B0 direction = [0, 0, 1]. The $\chi$-separation toolbox outputs a single negative (diamagnetic), positive (paramagnetic), and total *χ* map (QSM). The mean susceptibility of each subject’s SSS and CCV in their paramagnetic maps was calculated with the same vascular masks used for the QSM images. Sample images showing the magnitude, final QSM, and final paramagnetic component images are shown in Fig. 3.

Once the mean susceptibility values of the SSS and CCV were obtained from the subjects’ QSM images or paramagnetic maps, venous oxygen saturation (SvO_2_) was calculated with the following equation:^31^

1 $${{SvO}}_{2}=1-\frac{{\varDelta }_{{\chi }_{{blood}}}-\left({\varDelta }_{{\chi }_{{oxy}}}* {Hct}\right)}{{\varDelta }_{{\chi }_{{do}}* \,{Hct}}}$$

where $\varDelta {\chi }_{{blood}}$ is the vessel’s measured susceptibility, $\varDelta {\chi }_{{oxy}}$ is the constant representing the susceptibility changes of oxygenated blood relation to water, $\varDelta {\chi }_{{do}}$ is the difference in susceptibility between oxygenated and deoxygenated blood, and Hct is the subject’s hematocrit. $\varDelta {\chi }_{{oxy}}$ was −0.21 * 4$\pi$ ppm as per^32^ and,^33^ while $\varDelta {\chi }_{{do}}$ was −0.03 * 4$\pi$ ppm as per.^34^ Subjects’ Hct for the day of the scan was calculated using a four-parameter Weibull function with previously measured values while still in the NICU.

### Statistical analysis

Statistical analysis was performed using R and RStudio (v. 2023.09.1 Build 494).^35,36^ Mean and standard deviation values are reported for most statistics, unless specified otherwise. A paired Student’s t-test was used to determine statistical significance (*p* < 0.05) between two parameters (e.g., $\chi$ values between venous structures).

## Results

A total sample size of 19 infants were scanned, with a mean ($\pm$ standard deviation) gestational age of 28.8 $\pm$ 1.68 weeks and a mean birth weight of 1276.05 $\pm$ 294.87 grams. A comprehensive summary of neonatal characteristics, including additional demographic and clinical data, is provided in Table 2 for reference.

**Table 2:** Demographic and clinical characteristic of the study sample.

Variable	Subject data (*n* = 19)
Gestational age, weeks (mean ± SD)	28.8 ± 1.68
Corrected gestational age on scan day, weeks (mean ± SD)	40.36 ± 1.4
Number of male neonates (%)	10 (52.63)
Birth weight, g (mean ± SD)	1276.05 ± 294.87
Weight on scan day, g (mean ± SD)	3396.58 ± 597.72
Days spent in NICU (median, IQR)	53, 23
Days on ventilation (median, IQR)	31, 28.5

SD = standard deviation; IQR = inter quartile range.

The mean SvO_2_ values for the SSS and the CCV were found to be 0.72 $\pm$ 0.03% and 0.69 $\pm$ 0.03%, respectively, when determined from the QSM data. When determined from the paramagnetic map, the mean SvO_2_ values for the SSS and the CCV were found to be 0.58 $\pm$ 0.07% and 0.58 $\pm$ 0.07%, respectively. A summary of the measured physiological parameters, including the chi values used to calculate SvO_2_, can found in Table 3.

**Table 3:** Summary of acquired physiological parameters.

Region	Measure	QSM	Paramagnetic map	*p*-value	95% CI
SSS	Chi (ppm)	0.1 ± 0.02	0.21 ± 0.05	2.84e−11	−0.13, −0.09
SSS	SvO_2_ (%)	72.46 ± 3.43	58.14 ± 7.3	6.12e−10	0.12, 0.17
CCV	Chi (ppm)	0.13 ± 0.02	0.22 ± 0.05	6.25e−09	−0.1, −0.07
CCV	SvO_2_ (%)	68.71 ± 3.46	57.69 ± 6.97	2.16e−09	0.09, 0.13

Mean $\pm \,$SD is shown for chi and SvO_2_ values. The *P*-value and 95% confidence interval (CI) were obtained through the comparison of values between QSM and paramagnetic maps; (*n* = 19).

*QSM* quantitative susceptibility mapping, *CI* confidence interval, *SSS* superior sagitall sinus, *CCV* central cerebral vein.

**Fig. 4:**
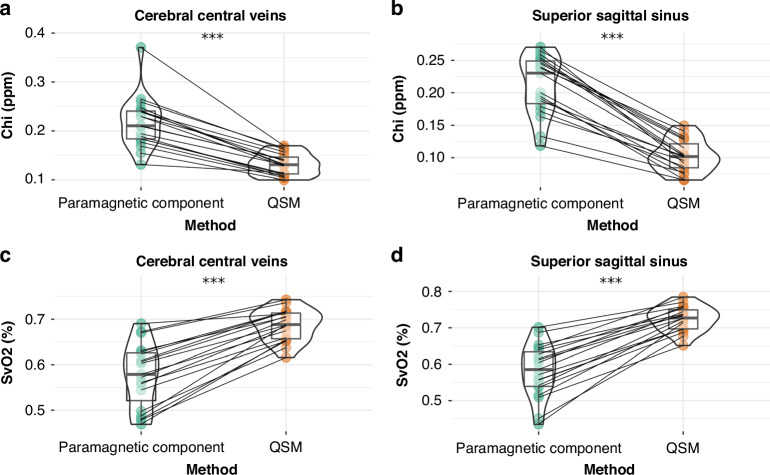
Vein-specific susceptibility and oxygen saturation values by method. **a**, **b** contains violin plots comparing subject chi (ppm) acquired from the cerebral central veins; **c**, **d** contains violin plots comparing subject SvO2 (%) acquired from the superior sagittal sinus. Raw data points from paramagnetic maps are shown as filled green circles, and raw data points from QSM are shown as filled orange circles. Each line connects the raw data points of a single subject. (***) indicates *P* < 0.05.

Region-specific $\chi$ and SvO_2_ values acquired from QSM were compared to values acquired from paramagnetic maps. In both the SSS and CCV, it was found that a significant difference existed between values acquired ($\chi$ and SvO_2_) from QSM and paramagnetic maps (*p* < 0.05). Violin plots of the comparisons are shown in Fig. 4.

**Fig. 5:**
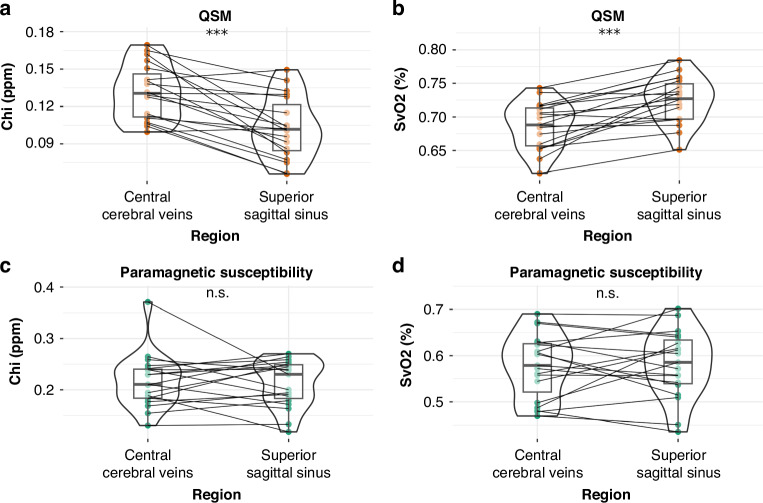
Inter-venous comparisons of susceptibility and oxygen saturation. Violin plots comparing (**a**, **c**) chi (ppm) and (**b**, **d**) SvO2 (%) between the CCV and the SSS. Panels (**a**) and (**b**) used data acquired from QSM, and its raw data points are shown as filled orange circles. Panels (**c**) and (**d**) used data acquired from paramagnetic maps, and its raw data points are shown as filled green circles. Each line connects the raw data points of a single subject. (***) indicates *p* < 0.05; (n.s.) indicates no significant difference.

The acquired $\chi$ and SvO_2_ values were additionally compared between veins. In data created from QSM, a significant difference was found between the CCV and SSS in mean $\chi$ (*p* < 0.05; 95% CI [0.017, 0.04]) and mean SvO_2_ (*p* < 0.05; 95% CI [−0.052, −0.023]). In data acquired from paramagnetic maps, no significant difference was observed between the CCV and the SSS in either mean $\chi$ (*p* = 0.711; 95% CI [−0.02, 0.029]) or mean SvO_2_ (*p* = 0.752; 95% CI [−0.034, 0.029]). A summary of these comparisons is represented in Fig. 5.

## Discussion

The primary objective of the present study was to assess whether the application of magnetic susceptibility separation to neonatal QSM data could provide more accurate SvO_2_ measurements without the need for an arbitrary threshold value. To the best of our knowledge, we are the first to test this in a neonatal cohort, as susceptibility separation has been typically evaluated as a method of imaging myelin and brain iron in adult subjects.^30,37^ Our results showed that the SvO_2_ values of the SSS and CCV obtained from susceptibility separation are significantly lower than the respective SvO_2_ values obtained from QSM alone. When our results were compared to the literature (see below), we found that our SSS SvO_2_ data from susceptibility separation agreed well with the findings of other studies measuring SvO_2_ of the SSS in similar subject populations. Conversely, the paramagnetic CCV SvO_2_ data saw less agreement with the existing literature than the corresponding data from QSM. However, there is reason to believe our paramagnetic CCV values may be accurate given their similarity to the paramagnetic SSS values and the limitations of the two studies that observed CCV SvO_2_. Additionally, it is important to note that our SvO_2_ measurements from susceptibility separation had greater variance than our measurements from QSM, indicating a limitation that should be addressed in future research. Overall, the present work demonstrates the promise of susceptibility separation as an MRI post-processing technique that can measure the oxygenation of the cerebral veins of infant subjects, a useful marker of regional oxygen consumption in the brain.

### Comparison with literature values

To evaluate the validity of our results, we compared the mean SvO_2_ values we obtained through QSM and susceptibility separation to the mean SvO_2_ values found by MRI studies investigating the oxygenation of the SSS or the CCV. Notably, the number of studies that measure the SvO_2_ of the CCV, or any of its individual veins, in infants is fairly lower than the number of studies investigating the oxygenation of the SSS. Our comparison is summarized in Table 4.

**Table 4:** Cerebral oxygenation values of neonates and fetuses in the literature.

Region	Study	Subjects	Method	SvO~2~(%)
Whole-brain	Skov et al.^6^	Preterm neonates (*n* = 9)	NIRS	53.4 ± 15.4
Whole-brain	Skov et al.^6^	Asphyxiated term neonates (*n* = 10)	NIRS	67.3 ± 9.4
Whole-brain	Altman et al. (1993)	Preterm and term neonates with HIE (*n* = 11)	PET	21.6 ± 21.0
SSS	Gou et al.^38^	Healthy neonates (*n* = 37)	MRI: TRUST	66.7 ± 4.9
SSS	Jiang et al.^16^	Healthy neonates (*n* = 4)	MRI: aTRUPC	64.8 ± 2.9
SSS	De Vis et al.^12^	PT-TEA neonates (*n* = 18)	MRI: T2-TRIR	52.0 ± 12.0
SSS	Yadav et al. (2020)	Late third trimester fetuses (*n* = 33)	MRI: Susceptometry	58.6 ± 4.8
SSS	**This study**	PT-TEA neonates *n* = 19	MRI: QSM	72.46 ± 3.43
SSS	**This study**	PT-TEA neonates *n* = 19	MRI: χ-separation	58.14 ± 7.3
CCV	Weber et al.^18^	Preterm neonates with HIE (*n* = 8)	MRI: QSM	72.2 ± 6.0
CCV	Weber et al.^18^	Healthy neonates (*n* = 8)	MRI: QSM	73.6 ± 2.8
CCV	Jiang et al.^16^	Healthy neonates (*n* = 4)	MRI: aTRUPC	70.2 ± 3.3
CCV	**This study**	PT-TEA neonates *n* = 19	MRI: QSM	68.71 ± 0.03
CCV	**This study**	PT-TEA neonates *n* = 19	MRI: χ-separation	57.69 ± 6.97

*PT-TEA* born preterm and scanned at term-equivalent age; late third trimester >35 weeks gestational age, *HIE* hypoxic-ischemic encephalopathy, *TRUST* T2-relaxation-under-spin tagging, *aTRUPC* accelerated T2-relaxation-under-phase-contrast; *T2-TRIR* T2-tissue-relaxation-inversion-recovery, *SWI* susceptibility weighted imaging.

As shown in Table 4, the infants observed in MRI studies investigating cerebral vein oxygenation noticeably differ in clinical status, with three studies involving healthy neonates,^16,18,38^ three studies (including the present study) involving preterm neonates,^12,18^ and one study involving late third trimester fetuses.^32^ In the studies involving healthy neonates, the SvO_2_ of the SSS fell within the range of 64.8–66.6%,^16,38^ while the SvO_2_ of the CCV fell within the range of 70.2–73.6%.^16,18^ Notably, the SvO_2_ value of the SSS we obtained from susceptibility separation (58.14%) was closest to values obtained from the studies involving late third trimester fetuses^39^ or pre-term neonates,^12^ each finding an SSS SvO_2_ value of 58.6% and 52.0%, respectively. It is important to note the difference in MRI modalities used to obtain these values. For their study,^39^ used MR susceptometry, which involves measuring the difference in phase between the chosen vessel and its background in imaging data from an SWI scanning sequence.^39^ In,^12^ the authors used T2-TRIR, which allowed them to determine the transverse relaxation rate of blood within the vessel, which can be used alongside hematocrit data to estimate SvO_2_. Additionally, the GA of infants scanned in our study ranged between 37 and 44 weeks, while the GA of the fetuses scanned in ref. ^39^ was ≥35 weeks, and the GA of infants scanned in^12^ ranged between 38 and 40 weeks. As such, our SSS SvO_2_ values found through susceptibility separation show promise given their similarity to the SvO_2_ values found by refs. ^39^ and ^12^ two studies that involved comparable subject populations and used considerably different methods.

Conversely, the SvO_2_ value of the CCV we obtained through QSM (68.71%) was closest to values from similar studies in the literature. In,^18^ QSM was used to measure an SvO_2_ of 71.5% in preterm neonates with HIE and an SvO_2_ of 73.6% in healthy neonates. In their study,^16^ also involved healthy neonates and obtained an SvO_2_ of 70.2% through an accelerated TRUPC sequence. In contrast, the SvO_2_ of the CCV we obtained through susceptibility separation was 57.69%. This disparity from the literature, however, may not undermine the value we obtained, as the study design of^18^ and^16^ may prevent their values from being representative of the study demographic. In,^18^ the authors utilized an arbitrary 0.15 ppm threshold and included all $\chi$ values above 0.15 when measuring the mean $\chi$ of the CCV, which potentially led to the introduction of $\chi$ from veins outside the CCV. In,^16^ the authors acquired their data from 4 subjects, a notably small sample size. Given the limitations of the existing literature and the similarity of the mean paramagnetic CCV SvO_2_ value (57.69%) to the mean paramagnetic SSS SvO_2_ value (58.14%), it is plausible that susceptibility separation provides more accurate measurements of oxygenation in both cortical and subcortical veins. One reason for this is due to its ability to mitigate partial volume effects, which are likely to contaminate other methods resulting in inaccurate CSvO_2_ values.^30^

Another notable distinction between our findings and those of the existing literature was that we observed no significant oxygenation difference between the SSS and the CCV when $\chi$ was derived from paramagnetic maps.^16^, the only other study that also measured SvO_2_ in both the SSS and CCV, observed significantly lower oxygenation in the SSS (64.8%) when compared to the CCV (70.2%). Given the small sample size utilized by,^16^ it is difficult to ascertain whether this is generalizable to all neonates.

### Limitations and future directions

This study has a few limitations that should be considered for future research. Firstly, only 19 infants were recruited for scanning. Given the emotional toll placed on parents when their child is born preterm, it is understandable that they may show reluctance in consenting to further testing that is not medically necessary. Obtaining a larger sample size in future studies, however, may provide greater insight into the efficacy of susceptibility separation. Secondly, this study did not include healthy neonates born at term, resulting in a lack of a control cohort. This is because recruiting healthy controls when there is no contraindication is very difficult. The addition of such a group may provide further validity to any findings and may reveal potential differences in cerebral oxygen consumption between term and preterm neonates. Finally, future studies should consider the use of multi-echo T2 imaging data when performing the decomposition of QSM images. The toolbox applied by this study for QSM decomposition^30^ utilizes R2 data, which can be obtained from multi-echo T2 imaging. Our study protocol involved the collection of multi-echo SWI imaging data, and as such, we could only use R2^*^ data to perform the decomposition. Furthermore, this may account for the reduced precision of SvO_2_ values obtained through susceptibility separation.

## Conclusion

This study aimed to evaluate how the use of susceptibility separation on preterm neonatal QSM images could be used in determining the oxygenation of cerebral venous vessels. We compared venous specific SvO_2_ values obtained from QSM images and their respective paramagnetic components to SvO_2_ values from neonatal MRI studies. We found that susceptibility separation provided SvO_2_ values of the SSS that were comparable to values found in the literature, providing evidence that this processing technique may be a valid tool for measuring regional cerebral oxygen consumption. Additionally, we were able to simultaneously measure SvO_2_ in both the SSS and CCV, which permitted us to observe no difference in oxygenation between the two vessels when considering data from isolated paramagnetic components. Ultimately, we hope our work inspires future studies that seek to explore and improve the capabilities of magnetic susceptibility separation, culminating in the development of a tool for clinicians and researchers alike.

## Data Availability

The manuscript was written in a ‘reproducible manner’. The entire manuscript, including statistics reported, figures, and tables, can be reproduced here: https://github.com/WeberLab/Chisep_CSVO2_Manuscript. Unfortunately, we can not upload our MRI images to an open repository as we did not obtain permission in our consent forms.
